# Supplementary material for: Link prediction accuracy on real-world networks under non-uniform missing-edge patterns
Source: PLoS One. 2024 Jul 18;19(7):e0306883. doi: 10.1371/journal.pone.0306883 (PMC11257260; doi:10.1371/journal.pone.0306883)
Supplement: S1 File — (PDF) [file pone.0306883.s001.pdf]

Supporting Information

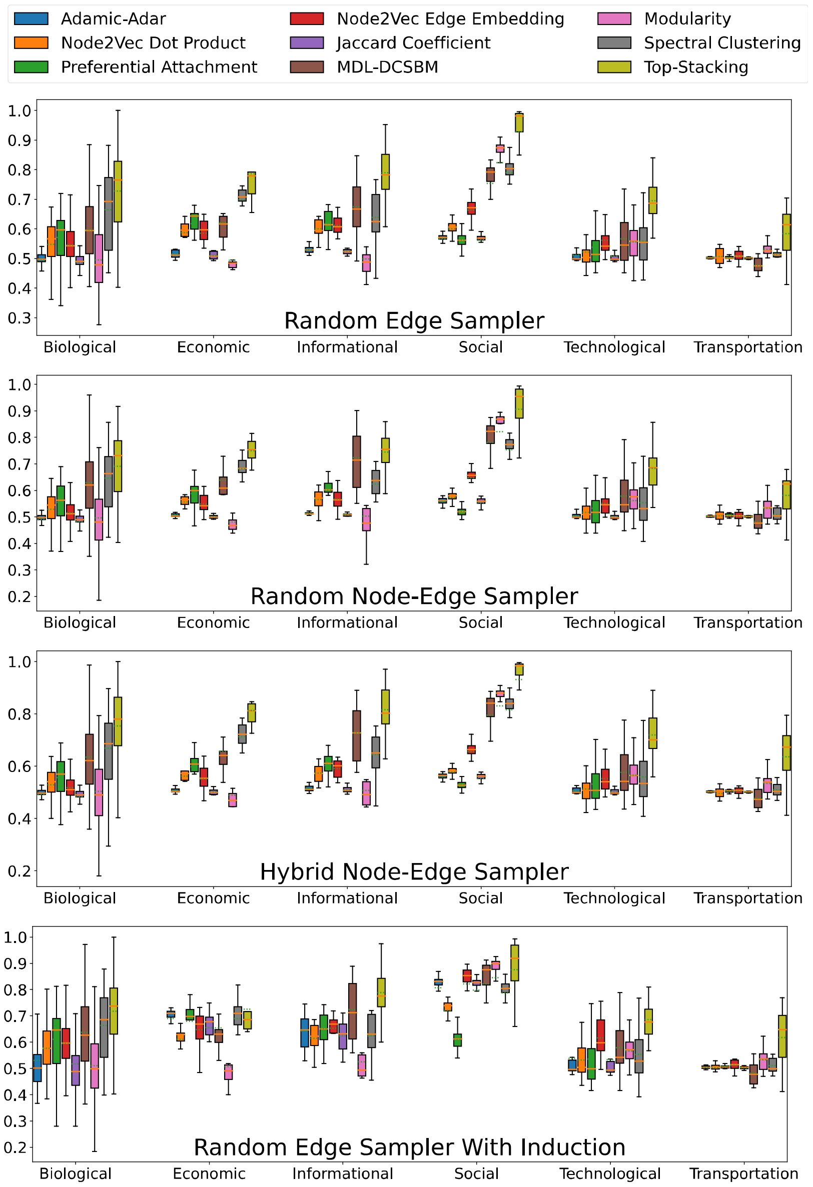

SI Figure 1. AUCs for Edge-Based Missingness Patterns from different link prediction methods, grouped by network domain, plotted as described in Fig 2 in the main text, with outliers removed for improved visibility.

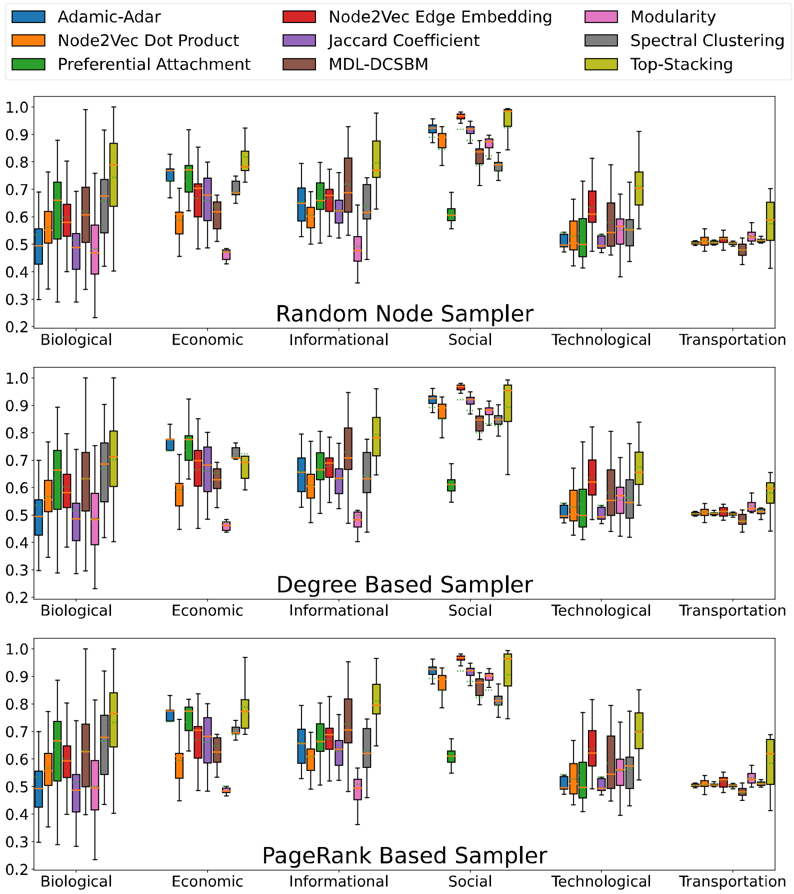

SI Figure 2. AUCs for Node-Based Missingness Patterns from different link prediction methods, grouped by network domain, plotted as described in Fig 2 in the main text, with outliers removed for improved visibility.

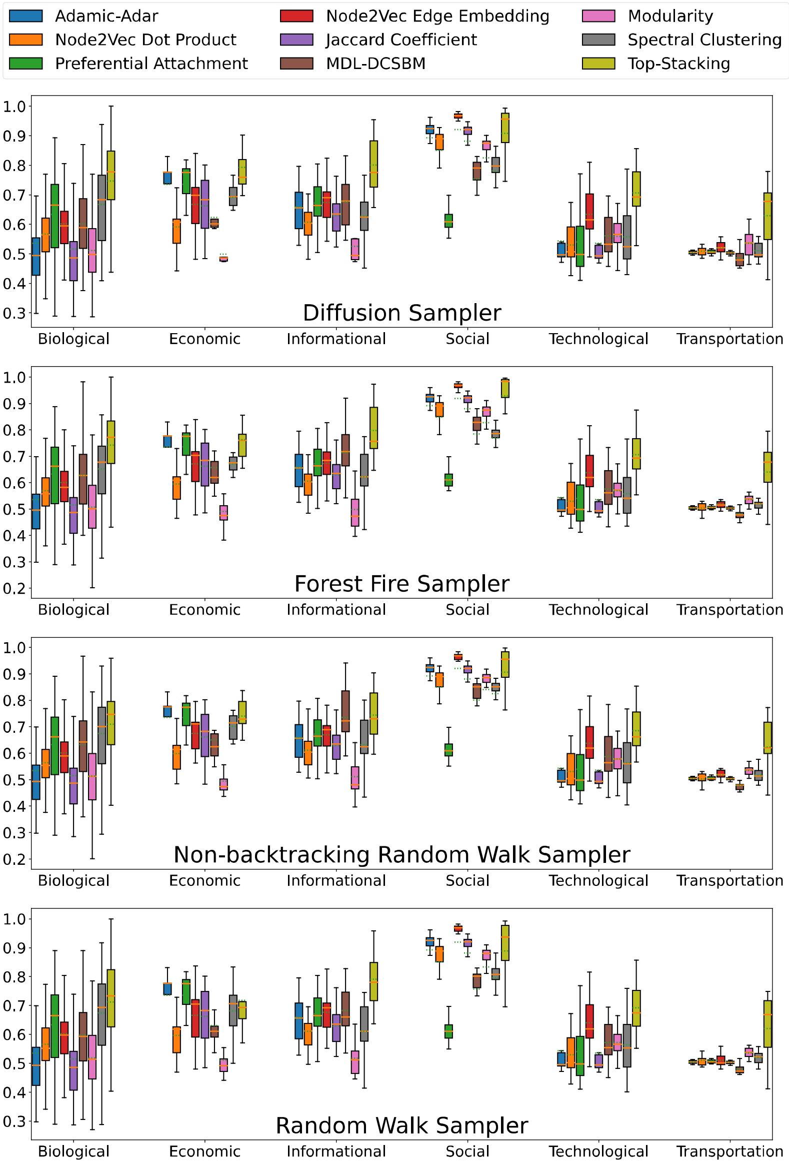

SI Figure 3. AUCs for Neighbor-Based Missingness Patterns from different link prediction methods, grouped by network domain (Part 1 of 2), plotted as described in Fig 2 in the main text, with outliers removed for improved visibility.

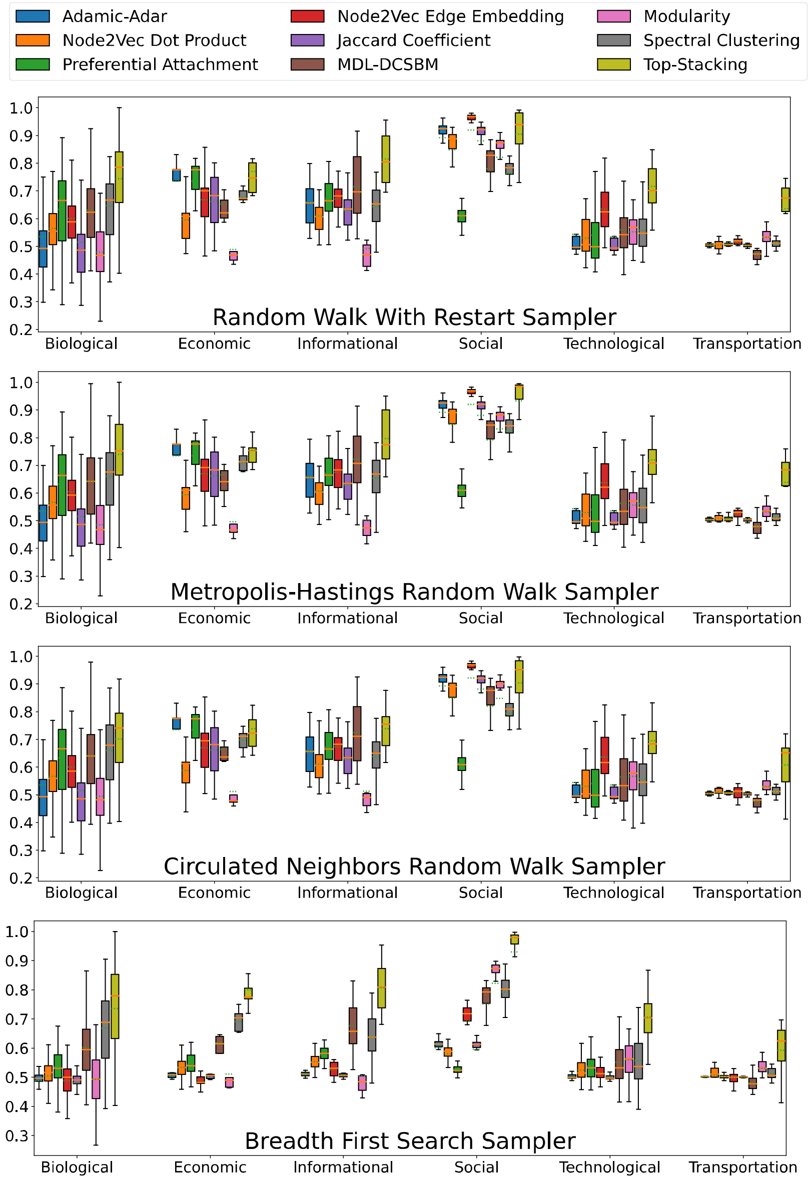

SI Figure 4. AUCs for Neighbor-Based Missingness Patterns from different link prediction methods, grouped by network domain (Part 2 of 2), plotted as described in Fig 2 in the main text, with outliers removed for improved visibility.

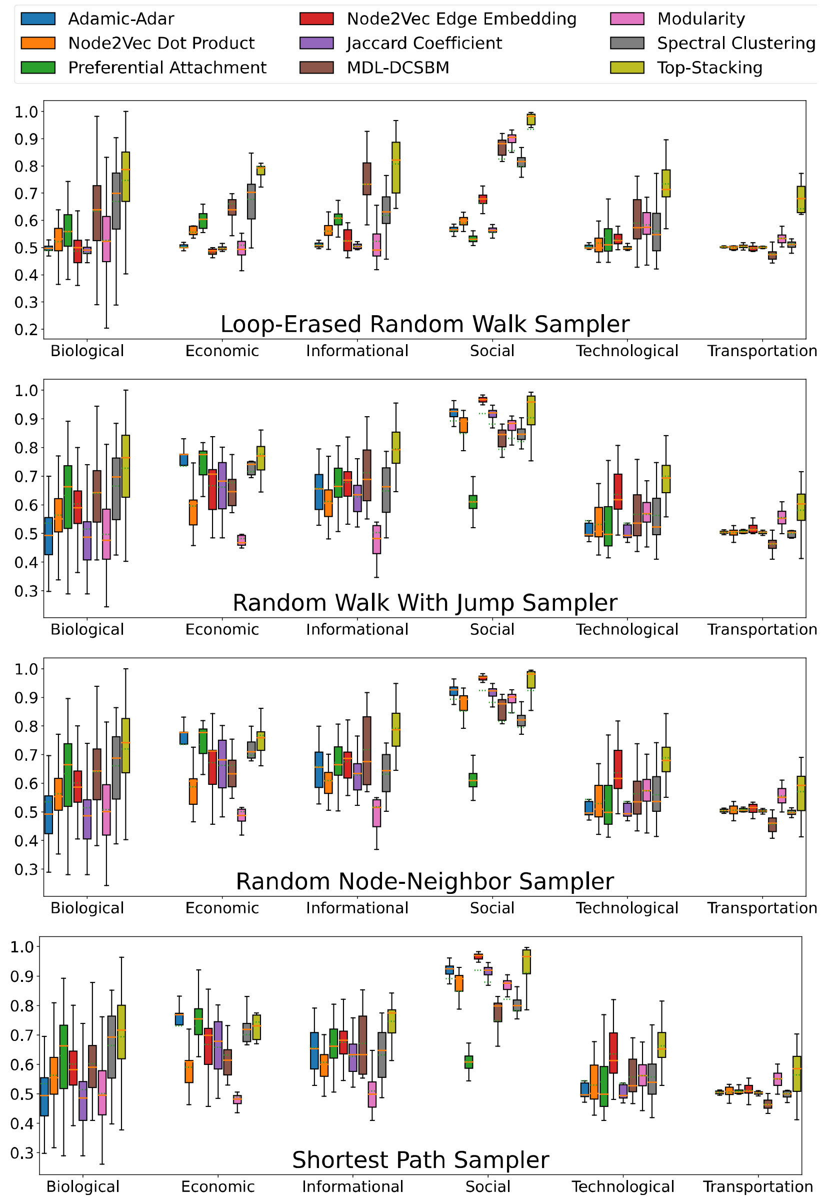

SI Figure 5. AUCs for Jump-Based Missingness Patterns from different link prediction methods, grouped by network domain, plotted as described in Fig 2 in the main text, with outliers removed for improved visibility.

SI Table 1. Average AUC scores over 5 runs of each of the biological networks.

Category	Missingness Pattern (Sampler)	Adamic-Adar	Node2Vec Dot Product	Prefer-ential Attachment	Node2Vec Edge Embedding	Jaccard Coefficient	MDL-DCSBM	Modularity	Spectral Clustering	Top-Stacking
Edge-Based	Random Edge Sampler	0.494	0.548	0.570	0.545	0.486	0.597	0.495	0.664	**0.728**
	Random Node-Edge Sampler	0.490	0.529	0.555	0.516	0.482	0.630	0.496	0.646	**0.690**
	Hybrid Node-Edge Sampler	0.491	0.528	0.556	0.518	0.483	0.635	0.503	0.669	**0.753**
	Random Edge Sampler With Induction	0.531	0.577	0.611	0.595	0.517	0.637	0.511	0.664	**0.718**
Node-Based	Random Node Sampler	0.535	0.562	0.627	0.599	0.515	0.621	0.483	0.657	**0.744**
	Degree Based Sampler	0.535	0.568	0.633	0.602	0.516	0.633	0.489	0.669	**0.702**
	PageRank Based Sampler	0.535	0.567	0.633	0.602	0.515	0.631	0.501	0.665	**0.733**
DFS	Depth First Search Sampler	0.501	0.523	0.518	0.496	0.492	0.630	0.489	0.654	**0.729**
Neighbor-Based	Diffusion Sampler	0.536	0.567	0.634	0.602	0.516	0.602	0.511	0.667	**0.747**
	Forest Fire Sampler	0.535	0.569	0.633	0.601	0.516	0.629	0.507	0.652	**0.742**
	Non-backtracking Random Walk Sampler	0.535	0.564	0.633	0.601	0.515	0.633	0.514	0.674	**0.709**
	Random Walk Sampler	0.535	0.566	0.634	0.603	0.515	0.598	0.519	0.674	**0.712**
	Random Walk With Restart Sampler	0.535	0.564	0.633	0.601	0.515	0.631	0.478	0.646	**0.743**
	Metropolis-Hastings Random Walk Sampler	0.535	0.566	0.633	0.604	0.516	0.642	0.485	0.660	**0.739**
	Circulated Neighbors Random Walk Sampler	0.535	0.567	0.632	0.600	0.515	0.641	0.494	0.661	**0.701**
	Breadth First Search Sampler	0.488	0.505	0.534	0.489	0.481	0.602	0.494	0.668	**0.736**
Jump-Based	Random Walk With Jump Sampler	0.535	0.564	0.633	0.603	0.515	0.640	0.497	0.665	**0.728**
	Random Node-Neighbor Sampler	0.535	0.563	0.633	0.602	0.514	0.640	0.508	0.661	**0.720**
	Shortest Path Sampler	0.534	0.563	0.630	0.601	0.515	0.603	0.502	0.664	**0.694**
	Loop-Erased Random Walk Sampler	0.489	0.522	0.560	0.486	0.481	0.634	0.527	0.669	**0.746**

SI Table 2. Average AUC scores over 5 runs of each of the economic networks.

Category	Missingness Pattern (Sampler)	Adamic-Adar	Node2Vec Dot Product	Preferential Attachment	Node2Vec Edge Embedding	Jaccard Coefficient	MDL-DCSBM	Modularity	Spectral Clustering	Top-Stacking
Edge-Based	Random Edge Sampler	0.515	0.588	0.618	0.584	0.510	0.622	0.494	0.698	**0.786**
	Random Node-Edge Sampler	0.508	0.556	0.588	0.549	0.502	0.639	0.481	0.682	**0.750**
	Hybrid Node-Edge Sampler	0.507	0.562	0.598	0.552	0.502	0.658	0.488	0.713	**0.813**
	Random Edge Sampler With Induction	0.689	0.610	0.679	0.648	0.656	0.655	0.507	0.690	**0.726**
Node-Based	Random Node Sampler	0.731	0.587	0.730	0.668	0.659	0.638	0.474	0.690	**0.818**
	Degree Based Sampler	**0.735**	0.592	**0.735**	0.665	0.662	0.652	0.474	0.715	0.723
	PageRank Based Sampler	0.735	0.594	0.735	0.669	0.662	0.648	0.501	0.688	**0.788**
DFS	Depth First Search Sampler	0.501	0.506	0.507	0.507	0.495	0.655	0.472	0.692	**0.738**
Neighbor-Based	Diffusion Sampler	0.735	0.591	0.736	0.667	0.662	0.623	0.499	0.691	**0.793**
	Forest Fire Sampler	0.735	0.595	0.736	0.671	0.662	0.657	0.491	0.670	**0.762**
	Non-backtracking Random Walk Sampler	0.735	0.599	0.736	0.674	0.662	0.650	0.494	0.701	**0.741**
	Random Walk Sampler	0.735	0.597	**0.736**	0.666	0.662	0.630	0.510	0.682	0.718
	Random Walk With Restart Sampler	0.735	0.596	0.736	0.668	0.662	0.653	0.489	0.678	**0.770**
	Metropolis-Hastings Random Walk Sampler	0.735	0.593	0.735	0.670	0.662	0.665	0.497	0.710	**0.755**
	Circulated Neighbors Random Walk Sampler	0.734	0.588	0.735	0.670	0.662	0.664	0.512	0.697	**0.739**
	Breadth First Search Sampler	0.507	0.534	0.543	0.490	0.502	0.619	0.511	0.691	**0.800**
Jump-Based	Random Walk With Jump Sampler	0.735	0.595	0.735	0.666	0.662	0.677	0.482	0.716	**0.777**
	Random Node-Neighbor Sampler	0.735	0.588	0.736	0.672	0.662	0.663	0.501	0.698	**0.767**
	Shortest Path Sampler	0.731	0.590	0.731	0.668	0.658	0.632	0.489	0.701	**0.743**
	Loop-Erased Random Walk Sampler	0.503	0.556	0.599	0.480	0.497	0.661	0.508	0.677	**0.797**

SI Table 3. Average AUC scores over 5 runs of each of the informational networks.

Category	Missingness Pattern (Sampler)	Adamic-Adar	Node2Vec Dot Product	Preferential Attachment	Node2Vec Edge Embedding	Jaccard Coefficient	MDL-DCSBM	Modularity	Spectral Clustering	Top-Stacking
Edge-Based	Random Edge Sampler	0.529	0.600	0.618	0.612	0.525	0.675	0.499	0.638	**0.790**
	Random Node-Edge Sampler	0.513	0.565	0.600	0.567	0.509	0.723	0.503	0.625	**0.745**
	Hybrid Node-Edge Sampler	0.515	0.573	0.603	0.591	0.511	0.727	0.508	0.643	**0.816**
	Random Edge Sampler With Induction	0.643	0.614	0.651	0.653	0.624	0.724	0.525	0.624	**0.788**
Node-Based	Random Node Sampler	0.653	0.605	0.664	0.664	0.624	0.719	0.492	0.637	**0.796**
	Degree Based Sampler	0.659	0.609	0.672	0.677	0.628	0.731	0.498	0.645	**0.794**
	PageRank Based Sampler	0.658	0.608	0.672	0.672	0.628	0.728	0.509	0.630	**0.810**
DFS	Depth First Search Sampler	0.502	0.506	0.510	0.504	0.497	0.713	0.493	0.632	**0.762**
Neighbor-Based	Diffusion Sampler	0.659	0.607	0.671	0.674	0.629	0.677	0.525	0.628	**0.801**
	Forest Fire Sampler	0.658	0.606	0.671	0.673	0.628	0.714	0.498	0.622	**0.798**
	Non-backtracking Random Walk Sampler	0.659	0.612	0.672	0.670	0.629	0.735	0.512	0.632	**0.744**
	Random Walk Sampler	0.659	0.611	0.671	0.676	0.628	0.683	0.518	0.611	**0.791**
	Random Walk With Restart Sampler	0.659	0.610	0.672	0.671	0.629	0.716	0.492	0.643	**0.814**
	Metropolis-Hastings Random Walk Sampler	0.658	0.607	0.671	0.675	0.628	0.722	0.503	0.653	**0.796**
	Circulated Neighbors Random Walk Sampler	0.659	0.610	0.672	0.673	0.628	0.725	0.513	0.638	**0.739**
	Breadth First Search Sampler	0.512	0.553	0.577	0.525	0.508	0.677	0.505	0.637	**0.807**
Jump-Based	Random Walk With Jump Sampler	0.658	0.611	0.672	0.677	0.628	0.711	0.503	0.648	**0.795**
	Random Node-Neighbor Sampler	0.659	0.609	0.672	0.676	0.628	0.717	0.518	0.631	**0.792**
	Shortest Path Sampler	0.657	0.607	0.669	0.674	0.628	0.671	0.510	0.632	**0.746**
	Loop-Erased Random Walk Sampler	0.510	0.561	0.605	0.526	0.505	0.733	0.523	0.619	**0.807**

SI Table 4. Average AUC scores over 5 runs of each of the social networks.

Category	Missingness Pattern (Sampler)	Adamic-Adar	Node2Vec Dot Product	Preferential Attachment	Node2Vec Edge Embedding	Jaccard Coefficient	MDL-DCSBM	Modularity	Spectral Clustering	Top-Stacking
Edge-Based	Random Edge Sampler	0.566	0.601	0.563	0.663	0.562	0.754	0.824	0.790	**0.928**
	Random Node-Edge Sampler	0.557	0.573	0.521	0.648	0.553	0.781	0.822	0.753	**0.906**
	Hybrid Node-Edge Sampler	0.558	0.578	0.531	0.655	0.554	0.795	0.831	0.816	**0.930**
	Random Edge Sampler With Induction	0.807	0.720	0.610	0.822	0.798	0.821	0.846	0.788	**0.876**
Node-Based	Random Node Sampler	0.890	0.845	0.610	0.919	0.879	0.787	0.822	0.769	**0.924**
	Degree Based Sampler	0.892	0.850	0.613	**0.921**	0.881	0.800	0.834	0.826	0.894
	PageRank Based Sampler	0.892	0.848	0.613	**0.919**	0.881	0.823	0.850	0.796	0.906
DFS	Depth First Search Sampler	0.514	0.523	0.504	0.556	0.510	0.775	0.821	0.763	**0.890**
Neighbor-Based	Diffusion Sampler	0.893	0.850	0.614	**0.921**	0.882	0.754	0.825	0.782	0.908
	Forest Fire Sampler	0.892	0.848	0.612	0.919	0.880	0.785	0.827	0.769	**0.929**
	Non-backtracking Random Walk Sampler	0.892	0.849	0.614	**0.921**	0.881	0.801	0.840	0.825	0.907
	Random Walk Sampler	0.893	0.851	0.614	**0.920**	0.882	0.757	0.833	0.796	0.889
	Random Walk With Restart Sampler	0.892	0.848	0.613	**0.919**	0.881	0.780	0.821	0.763	0.904
	Metropolis-Hastings Random Walk Sampler	0.892	0.849	0.613	0.920	0.881	0.792	0.832	0.824	**0.933**
	Circulated Neighbors Random Walk Sampler	0.893	0.850	0.614	**0.921**	0.881	0.820	0.849	0.795	0.905
	Breadth First Search Sampler	0.601	0.581	0.527	0.692	0.596	0.751	0.822	0.792	**0.929**
Jump-Based	Random Walk With Jump Sampler	0.892	0.849	0.613	**0.918**	0.881	0.793	0.832	0.820	0.904
	Random Node-Neighbor Sampler	0.894	0.852	0.613	0.923	0.882	0.816	0.847	0.796	**0.924**
	Shortest Path Sampler	0.891	0.848	0.611	**0.920**	0.880	0.748	0.821	0.783	0.911
	Loop-Erased Random Walk Sampler	0.560	0.590	0.536	0.663	0.555	0.825	0.856	0.798	**0.935**

SI Table 5. Average AUC scores over 5 runs of each of the technological networks.

Category	Missingness Pattern (Sampler)	Adamic-Adar	Node2Vec Dot Product	Preferential Attachment	Node2Vec Edge Embedding	Jaccard Coefficient	MDL-DCSBM	Modularity	Spectral Clustering	Top-Stacking
Edge-Based	Random Edge Sampler	0.511	0.521	0.538	0.561	0.507	0.566	0.556	0.560	**0.695**
	Random Node-Edge Sampler	0.505	0.518	0.533	0.552	0.501	0.578	0.562	0.553	**0.684**
	Hybrid Node-Edge Sampler	0.506	0.513	0.531	0.553	0.503	0.577	0.565	0.559	**0.720**
	Random Edge Sampler With Induction	0.539	0.532	0.535	0.616	0.532	0.579	0.566	0.553	**0.686**
Node-Based	Random Node Sampler	0.542	0.528	0.538	0.634	0.534	0.579	0.551	0.559	**0.712**
	Degree Based Sampler	0.544	0.529	0.540	0.636	0.536	0.582	0.554	0.565	**0.675**
	PageRank Based Sampler	0.544	0.528	0.540	0.637	0.536	0.585	0.556	0.563	**0.702**
DFS	Depth First Search Sampler	0.501	0.503	0.501	0.508	0.497	0.561	0.560	0.559	**0.669**
Neighbor-Based	Diffusion Sampler	0.544	0.530	0.541	0.634	0.536	0.561	0.559	0.557	**0.707**
	Forest Fire Sampler	0.544	0.530	0.540	0.638	0.535	0.576	0.570	0.557	**0.706**
	Non-backtracking Random Walk Sampler	0.544	0.530	0.540	0.637	0.536	0.588	0.578	0.564	**0.685**
	Random Walk Sampler	0.544	0.530	0.541	0.640	0.536	0.572	0.572	0.563	**0.692**
	Random Walk With Restart Sampler	0.544	0.529	0.540	0.634	0.535	0.559	0.553	0.559	**0.717**
	Metropolis-Hastings Random Walk Sampler	0.544	0.529	0.540	0.637	0.536	0.563	0.556	0.562	**0.720**
	Circulated Neighbors Random Walk Sampler	0.544	0.531	0.540	0.636	0.535	0.564	0.567	0.557	**0.695**
	Breadth First Search Sampler	0.504	0.524	0.539	0.517	0.501	0.551	0.559	0.557	**0.706**
Jump-Based	Random Walk With Jump Sampler	0.544	0.531	0.541	0.636	0.536	0.567	0.566	0.563	**0.700**
	Random Node-Neighbor Sampler	0.544	0.529	0.540	0.637	0.536	0.564	0.572	0.560	**0.690**
	Shortest Path Sampler	0.544	0.530	0.540	0.635	0.536	0.557	0.562	0.557	**0.663**
	Loop-Erased Random Walk Sampler	0.504	0.516	0.537	0.529	0.500	0.590	0.584	0.560	**0.734**

SI Table 6. Average AUC scores over 5 runs of each of the transportation networks.

Category	Missingness Pattern (Sampler)	Adamic-Adar	Node2Vec Dot Product	Preferential Attachment	Node2Vec Edge Embedding	Jaccard Coefficient	MDL-DCSBM	Modularity	Spectral Clustering	Top-Stacking
Edge-Based	Random Edge Sampler	0.503	0.507	0.503	0.509	0.502	0.476	0.531	0.515	**0.583**
	Random Node-Edge Sampler	0.502	0.505	0.506	0.504	0.501	0.483	0.533	0.510	**0.581**
	Hybrid Node-Edge Sampler	0.502	0.496	0.505	0.504	0.501	0.478	0.534	0.510	**0.635**
	Random Edge Sampler With Induction	0.504	0.502	0.507	0.512	0.503	0.482	0.533	0.511	**0.617**
Node-Based	Random Node Sampler	0.504	0.513	0.508	0.517	0.503	0.478	0.530	0.512	**0.575**
	Degree Based Sampler	0.504	0.510	0.507	0.511	0.503	0.480	0.533	0.516	**0.580**
	PageRank Based Sampler	0.504	0.507	0.508	0.517	0.503	0.478	0.532	0.513	**0.585**
DFS	Depth First Search Sampler	0.500	0.508	0.504	0.500	0.499	0.458	0.551	0.505	**0.575**
Neighbor-Based	Diffusion Sampler	0.504	0.509	0.508	0.519	0.503	0.486	0.536	0.510	**0.629**
	Forest Fire Sampler	0.504	0.503	0.508	0.520	0.503	0.482	0.536	0.520	**0.640**
	Non-backtracking Random Walk Sampler	0.504	0.502	0.508	0.518	0.503	0.478	0.535	0.518	**0.623**
	Random Walk Sampler	0.504	0.510	0.508	0.510	0.503	0.480	0.536	0.520	**0.621**
	Random Walk With Restart Sampler	0.504	0.505	0.508	0.515	0.503	0.473	0.532	0.512	**0.636**
	Metropolis-Hastings Random Walk Sampler	0.504	0.508	0.507	0.524	0.503	0.477	0.531	0.511	**0.639**
	Circulated Neighbors Random Walk Sampler	0.504	0.511	0.509	0.508	0.503	0.477	0.530	0.512	**0.608**
	Breadth First Search Sampler	0.502	0.507	0.503	0.499	0.501	0.480	0.530	0.511	**0.593**
Jump-Based	Random Walk With Jump Sampler	0.504	0.503	0.508	0.517	0.503	0.463	0.556	0.500	**0.581**
	Random Node-Neighbor Sampler	0.504	0.505	0.508	0.507	0.503	0.457	0.555	0.499	**0.571**
	Shortest Path Sampler	0.504	0.506	0.508	0.512	0.503	0.465	0.550	0.501	**0.564**
	Loop-Erased Random Walk Sampler	0.502	0.500	0.504	0.497	0.501	0.476	0.535	0.513	**0.643**
