# Supplementary material for: Link prediction accuracy on real-world networks under non-uniform missing-edge patterns
Source: PLoS One. 2024 Jul 18;19(7):e0306883. doi: 10.1371/journal.pone.0306883 (PMC11257260; doi:10.1371/journal.pone.0306883)
Supplement: Attachment — Submitted filename: PLOS_ONE_Sampling_Response_letter_Revision_1.pdf [file pone.0306883.s002.pdf]


Review 1

R1.1

This manuscript validates link prediction methods with empirical data and a variety of methods to select and hide edges. It is an important reference work for network science and graph learning. The manuscript is clear and well-written. The methods are sound. There is no reason to request a revision, it could be published as it is.

If anything should be polished: 1. The box-and-whiskers plots have too many outliers. Sometimes, the outliers distract the viewer from seeing the patterns shown by the boxes. These could either be plotted without outliers or replaced by other visualization techniques. 2. Trellis plots like Fig. 3 should have the same limits on the axes for readers to compare the panels efficiently.

We really appreciate your affirmation of our work and the thoughtful comments about the plots.

We have revised Figure 2 and Supplementary Figures S1–S5 to remove the outliers and noted in the captions that this has been done to improve visibility.

Regarding Figure 3, while we understand and agree that unified limits could be helpful, the significant differences between the plots make it challenging to implement in a way that maintains good visibility. Since unifying the limits would obscure the distinctions between some methods, we have decided to retain the current scaling to ensure clarity.

Thank you again for your supportive comments about our work.

Review 2

We thank the reviewer for their comments, which we have organized below into distinct points, copying the totality of the review (in italics), followed by our response to each point (in plain text). We note that additions to the manuscript in response to the reviews appears in blue.

R2.1

The author indeed aims to extract the applicability of several classic complex network link prediction algorithms from a vast dataset, further elucidating the underlying mechanism of missing edges. However, the article lacks depth in both physical understanding and computational thoroughness. Firstly, the rationale for network sampling is questionable; if it was to save time, this is unlikely to convince readers. While the author attempts to explain, it fails to ensure that the structural features of the sampled network resemble those of the original, instead resorting to various computational strategies, employing numerous sampling techniques to compensate for technical shortcomings. Paradoxically, the more techniques employed, the murkier the underlying principles become, leading to a superficial analysis.

We are grateful for the reviewer's comments as an opportunity for us to clarify important items of our manuscript to avoid possible misunderstandings regarding the purpose and use of sampling in this paper. Since most network data sets are inherently sampled approximations of the full set of connections present in the real world, our purpose in employing different sampling methods is to mimic various possible missingness patterns that might arise in real-world data collection and to demonstrate and evaluate how different link prediction algorithms perform under these different conditions. Our present aim is not to save time (though in some settings, researchers

performing network surveys do attempt link prediction as a more efficient and expedient means for overcoming missing data as opposed to further surveying). To avoid this misunderstanding of our purpose, we have edited the first paragraph of our Introduction to endeavor to make this clearer (with new text highlighted in blue):

> Link prediction serves as a valuable tool to correct for missingness inherent in most methods of network data collection. Indeed, many network datasets are only partial samples of the full set of connections present in the real world and, as such, link prediction is a prevalent technique in network analysis...

We believe the subsequent statements the reviewer makes about "technical shortcomings" to also be addressed by such clarification in that the numerous sampling methods we intentionally used were to mimic diverse types of missing patterns that might occur in real-world scenarios, as discussed in the (unchanged) second paragraph of the Introduction. That is, it is a deliberate strategy on our part to employ these multiple techniques and to then comprehensively evaluate the differences in the accuracies of different link prediction algorithms for data from different disciplinary domains under these different sampling conditions.

R2.2

Secondly, I note the low predictive accuracy for technological and transportation networks, which I suspect relates to network sparsity. References to 'Shang, K. K., Li, T. C., Small, M., Burton, D., Wang, Y. (2019). Link prediction for tree-like networks. Chaos: An Interdisciplinary Journal of Nonlinear Science, 29(6); and Shang, K. K., Small, M. (2022). Link prediction for long-circle-like networks. Physical Review E, 105(2), 024311' might provide insight.

We appreciate this comment about the observed performance on technological networks and transportation networks, and have added the suggested references coauthored by the reviewer to a short discussion on this observation in our results section on Page 9:

> We note that the link prediction accuracies are typically notably less accurate for technological and transportation networks across sampling methods and link prediction algorithms compared to accuracies for networks from the other four disciplinary domains. We suspect two possible reasons for this. First, the limited number of these types of networks in our dataset collection might bias the results, though in hypothesizing such an effect we note that the numbers of networks we study from the economic and informational domains are also small. Perhaps more likely, there is some systematic network feature or properties about data from these domains that could be found to be related to lower link prediction accuracy. As one for example, we note that performing link prediction in very sparse networks can lead to additional challenges (see, e.g., [55], [56]).

R2.3

Moreover, since 2019, there has been a surge in utilizing graph theory combined with machine learning and neural networks for link prediction, hence the inclusion of such contemporary algorithms is recommended.

We agree that neural networks are becoming increasingly popular for many applications and have added a clarifying discussion about this limitation of our study on Pages 13–14. Nevertheless, we note that neural network methods were not included because our primary goal is to compare the

overall impact on commonly used and available link prediction methods under different sampling procedures and across different disciplinary domains. While we recognize that neural network methods can deliver outstanding classification performance in many settings, we are unaware of any specific method for link prediction appropriate to the present task, specifically, without the need for any additional node-level metadata, that meets our requirement that the algorithm exist in publicly available code. Moreover, and importantly, we are unaware of any such method providing consistently good performance at or beyond that of the "Top-Stacking" method we use here. As an additional benefit, all of the methods considered here have some interpretability, providing some understanding of the underlying features driving those predictions and thus providing better explainability. Nevertheless, we acknowledge this limitation in our revised Discussion:

> Neural network methods have also been successfully used for link prediction to recover missing edges [59]. However, recent publications [25,60] have raised concerns about their performance relative to more traditional algorithms, as well as their lack of feature explainability, which poses challenges in understanding their performance. While we recognize that neural network methods can deliver outstanding classification performance in many settings, we are unaware of any specific neural network methods for link prediction appropriate to the present task, specifically, without the need for any additional node-level metadata, that exists in publicly available code, and that focuses on feature explainability. Consequently, this study excludes neural network methods and instead prioritizes the understanding of behaviors and features beneficial for diverse real-world applications. Nevertheless, we acknowledge the limitation that, in comparing the overall impact on the accuracy of different link prediction algorithms under different sampling settings and across different domains, an appropriate neural network method might provide a different pattern of variation in these observed accuracies. Conducting a comprehensive examination of neural network methods and comparing them could provide valuable insights for future research.

R2.4

Proceeding through the article, I observe a glaring absence of a comprehensive and meticulous differentiation of the mechanisms behind missing links in networks. A tabular summary to address this gap is advisable.

We think the reviewer's suggestion to include a tabular summary to be interesting and we subsequently considered it seriously. However, we were unable to identify a clean format for such a table that we thought would provide useful information to the reader above and beyond our grouping of these 20 methods into 5 different categories and the text summaries of these 20 methods that we already provide in detail across nearly two full pages of the manuscript (pp. 4–5). Moreover, as we note in the manuscript, all of these sampling methods are part of the Little Ball of Fur python library, and as such we believe the reader already has easy access to that documentation if they are interested in the meticulous details of each sampling method.

R2.5

Lastly, the choice to independently calculate the Area Under the Curve (AUC) only five times is puzzling, given that since the advent of link prediction techniques, 100 independent calculations have become a standard requirement to ensure statistical robustness.

We understand that in many cases in the literature it is standard procedure to include more than

5 calculations and, indeed, our results analyzed here include many more than 5 calculations when collected over the number of different networks considered in each disciplinary domain. Nevertheless, we acknowledge that this is a limitation of our study. However, given the comprehensive nature of our numerical experiments, we remain limited to these results. In particular, performing this study on the 250 networks comparing 20 different sampling methods and 9 different link prediction algorithms each performed 5 times encompasses 225,000 independent calculations totaling $\sim 10^5$ CPU core hours (approximately a month running in parallel on our cluster). Given some reasonable limitation on our computational resources, conducting only five trials per network-by-sampler-by-predictor was feasible within our constraints. We have acknowledged this limitation on Page 3 with new text:

> While conducting a larger number of independent trials would be ideal, we note that running the present suite of numerical experiments, sampling 250 datasets across 20 different missingness patterns and employing 9 link prediction algorithms, requires approximately 10^5 CPU core hours. Emphasizing that our reported results are at the disciplinary domain level, not the individual network level, our calculations yield 50 independent AUC scores for each of the smallest domains (economic and transportation).
